# Diagnostic accuracy of dynamic contrast‐enhanced perfusion MRI in stratifying gliomas: A systematic review and meta‐analysis

**DOI:** 10.1002/cam4.2369

**Published:** 2019-08-07

**Authors:** Sachi Okuchi, Antonio Rojas‐Garcia, Agne Ulyte, Ingeborg Lopez, Jurgita Ušinskienė, Martin Lewis, Sara M Hassanein, Eser Sanverdi, Xavier Golay, Stefanie Thust, Jasmina Panovska‐Griffiths, Sotirios Bisdas

**Affiliations:** ^1^ Department of Brain Repair and Rehabilitation Institute of Neurology, University College London London UK; ^2^ Department of Applied Health Research University College London London UK; ^3^ Epidemiology, Biostatistics and Prevention Institute University of Zurich Zurich Switzerland; ^4^ Neuroradiology, Institute of Neurosurgery Dr. A. Asenjo Santiago Chile; ^5^ Diagnostic and Interventional Radiology Department, Faculty of Medicine, National Cancer Institute Vilnius University Vilnius Lithuania; ^6^ Diagnostic Radiology Department, Faculty of Medicine Assiut University Assiut Egypt; ^7^ Lysholm Department of Neuroradiology National Hospital for Neurology and Neurosurgery London UK

**Keywords:** dynamic contrast‐enhanced MRI, gliomas, lymphoma, meta‐analysis, perfusion

## Abstract

**Background:**

T1‐weighted dynamic contrast‐enhanced (DCE) perfusion magnetic resonance imaging (MRI) has been broadly utilized in the evaluation of brain tumors. We aimed at assessing the diagnostic accuracy of DCE‐MRI in discriminating between low‐grade gliomas (LGGs) and high‐grade gliomas (HGGs), between tumor recurrence and treatment‐related changes, and between primary central nervous system lymphomas (PCNSLs) and HGGs.

**Methods:**

We performed this study based on the Preferred Reporting Items for Systematic Reviews and Meta‐Analysis of Diagnostic Test Accuracy Studies criteria. We systematically surveyed studies evaluating the diagnostic accuracy of DCE‐MRI for the aforementioned entities. Meta‐analysis was conducted with the use of a random effects model.

**Results:**

Twenty‐seven studies were included after screening of 2945 possible entries. We categorized the eligible studies into three groups: those utilizing DCE‐MRI to differentiate between HGGs and LGGs (14 studies, 546 patients), between recurrence and treatment‐related changes (9 studies, 298 patients) and between PCNSLs and HGGs (5 studies, 224 patients). The pooled sensitivity, specificity, and area under the curve for differentiating HGGs from LGGs were 0.93, 0.90, and 0.96, for differentiating tumor relapse from treatment‐related changes were 0.88, 0.86, and 0.89, and for differentiating PCNSLs from HGGs were 0.78, 0.81, and 0.86, respectively.

**Conclusions:**

Dynamic contrast‐enhanced‐Magnetic resonance imaging is a promising noninvasive imaging method that has moderate or high accuracy in stratifying gliomas. DCE‐MRI shows high diagnostic accuracy in discriminating between HGGs and their low‐grade counterparts, and moderate diagnostic accuracy in discriminating recurrent lesions and treatment‐related changes as well as PCNSLs and HGGs.

## Graphical abstract

Dynamic contrast‐enhanced‐Magnetic resonance imaging has high diagnostic accuracy in grading gliomas and moderate accuracy in surveillance of high‐grade gliomas and differentiation from primary central nervous system lymphomas. DCE‐MRI is a promising noninvasive imaging technique in diagnosing different types of brain tumors.

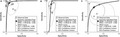

## 1 INTRODUCTION

Gliomas account for approximately 28% of all central nervous system tumors and 80% of all malignant brain tumors.^1^ The 2016 World Health Organization classification divides gliomas into grade I to IV, with grades I and II considered to be low‐grade gliomas (LGGs) and grades III and IV considered high‐grade gliomas (HGGs), on the basis of their histology and molecular features.^2^ Primary central nervous system lymphoma (PCNSL) most commonly occurs in the elderly^3^ and comprises 2.1% of primary intracranial tumors.^1^

The treatment options and prognosis are heavily dependent on the histological types and the recurrence status. The present standard therapy of HGGs is surgical resection and concomitant chemoradiation.^4^ Chemoradiation may knowingly result in radiation necrosis and pseudoprogression, which may notoriously resemble recurrence and tumor progression.^5^ Therefore, it is crucially important to utilize a noninvasive imaging technique that can differentiate them for the patient management.

Although magnetic resonance imaging (MRI) is routinely applied to classify brain tumors, conventional MRI has shortcomings.^6^, ^7^, ^8^, ^9^ To overcome such limitations, previous studies have reported combining conventional MRI with multimodal techniques, which increase the diagnostic accuracy.^9^, ^10^, ^11^

Perfusion‐weighted imaging is commonly used for the assessment and classification of intracranial tumors, and may be performed as dynamic susceptibility contrast‐enhanced (DSC) MRI, T1‐based dynamic contrast‐enhanced (DCE) MRI, and arterial spin labeling (ASL).^12^, ^13^, ^14^, ^15^ The most common MR perfusion technique in clinical practice is DSC‐MRI.^8^, ^12^ However, DCE‐MRI has added benefits of higher spatial resolution, more reliable quantification measurement of microvasculature and permeability indices, and reduced susceptibility artifacts with respect to DSC‐MRI.^16^, ^17^

A number of single‐center studies in mainly small cohorts have shown the potential of DCE‐MRI.^18^, ^19^, ^20^ Our work extends previous studies with a systematic large‐scale meta‐analysis and aims at evaluating the diagnostic value of DCE‐MRI. To achieve these aims, we have specifically explored whether using DCE measurements can successfully differentiate LGGs from HGGs, tumor recurrence from treatment‐related changes, and PCNSLs from HGGs.

## 2 MATERIALS AND METHODS

This study was performed in accordance with the Preferred Reporting Items for Systematic Reviews and Meta‐Analysis of Diagnostic Test Accuracy Studies criteria.^21^ This systematic review was registered in the PROSPERO online database of systematic reviews (CRD42018108948).

### 2.1 Search strategy

The search was systematically conducted on June 8, 2017 using PubMed, Ovid Embase, and the Cochrane Library. The detail of the search strategy is presented in the Supplementary Material 1.

### 2.2 Selection criteria

The abstracts of all articles retrieved in the initial search were screened by board‐certified neuroradiologists and in‐training neuroradiologists with research experience in perfusion imaging in neuro‐oncology. Selected full text manuscripts were reviewed to determine their relevance in detail. Both processes were executed by independent reviewers according to the following criteria. The inclusion criteria were: (a) DCE performed on brain tumor patients prior or during treatment; (b) study assessed diagnostic or prognostic value of DCE parameters. The exclusion criteria were: (a) no DCE (T1‐weighted perfusion) was performed; (b) no brain tumor patients were examined; (c) the study was conducted in pediatric population (<18 years old); (d) animal/laboratory study; (e) review articles, case reports, letters, commentaries, or conference proceedings; (f) brain tumor histology was not confirmed; (g) non‐English articles. In cases of discrepancies between two reviewers, a third one resolved the case.

For the meta‐analysis, selected full manuscripts were reviewed by two independent reviewers and in cases of discrepancies between two reviewers, all discrepancies were resolved by consensus. The inclusion criteria were: (a) the studies assessed the diagnostic accuracy of DCE‐MRI for discriminating between HGGs and LGGs, between recurrence and treatment‐related changes, and between PCNSLs and HGGs. The exclusion criteria were: (a) patient population clearly overlapped with other studies cohorts; (b) the information for extracting or calculating true‐negative (TN), false‐negative (FN), true‐positive (TP), and false‐positive (FP) values was not listed. Studies assessing the prognostic value of DCE‐MRI were excluded due to their small number. If overlapping studies showed no distinct information, the study with more patients was chosen.

### 2.3 Data extraction

Data were extracted from the included studies. Data included sensitivity and specificity to calculate subsequently the TN, FN, TP, and FP for each of the diagnostic task under investigation, number of patients, age of patients, study design, tumor histology, MRI field strength, whether DCE‐MRI was followed with DSC‐MRI or not, methods of a region of interest (ROI) analysis, deconvolution with arterial input function, and DCE model. The same two reviewers, who performed full‐text screening, independently conducted data extraction, and all inconsistencies were resolved by consensus.

### 2.4 Study quality assessment

We assessed the study quality based on the Quality Assessment of Diagnostic Accuracy Studies (QUADAS‐2) instrument (see Supplementary Material 2).^22^ Each study was evaluated for potential bias and quality by two independent reviewers experienced in neuro‐oncological imaging and advanced MRI techniques. Disagreements were resolved by consensus.

### 2.5 Statistical analysis

True‐negative, FN, TP, and FP values were calculated from the number of patients, and their sensitivity and specificity for statistical analysis. Two studies showed complete patients data but did not present calculations of sensitivity and specificity.^23^, ^24^ Therefore, we calculated these from the published patient data in each article using commercially available (MedCalc version 18.5 for Windows) software (Ostend, Belgium). Our statistical analysis explored the diagnostic accuracy of DCE in the following comparisons: HGGs vs LGGs, recurrence vs treatment‐related changes, and PCNSLs vs HGGs. Specifically, DTA meta‐analysis, subgroup analysis, heterogeneity, and publication bias were executed with the use of the MIDAS in STATA 15.0 (College Station, TX).

In DTA meta‐analysis, the pooled sensitivity, specificity, positive likelihood ratios (PLRs), negative likelihood ratios (NLRs), diagnostic odds ratios (DORs), and their 95% CIs were calculated for each comparison. The values of DCE parameters with the highest diagnostic accuracy were used. Random effects models were applied to address the expected heterogeneity. The accuracy was determined using a summary receiver operating characteristic curve (SROC) plot. To quantify error and accuracy, the area under the curve (AUC) was calculated. AUC values of more than 0.9 represented high accuracy and 0.7 ≤ AUC ≤ 0.9 reflected moderate accuracy.^25^

The pooled sensitivity and specificity were calculated in subgroups (studies number ≥4) created based on DCE perfusion imaging derived parameters (*K* ^trans^, v_e_, and v_p_), applied pharmacokinetic model (model‐independent and two‐compartment model approaches), and methods of ROI analysis (whole lesion volume, lesion “hot‐spot,” and operator‐selected tumor part).

Heterogeneity was tested with the use of the quantity *I* ^2^. An *I* ^2^ >50% indicated substantial heterogeneity. The publication bias was evaluated for the analyses including >10 studies^26^ with the use of funnel plot asymmetry test.^27^, ^28^ *P* < 0.10 indicated significant asymmetry and low publication bias.^27^, ^28^

## 3 RESULTS

**Figure 1:**
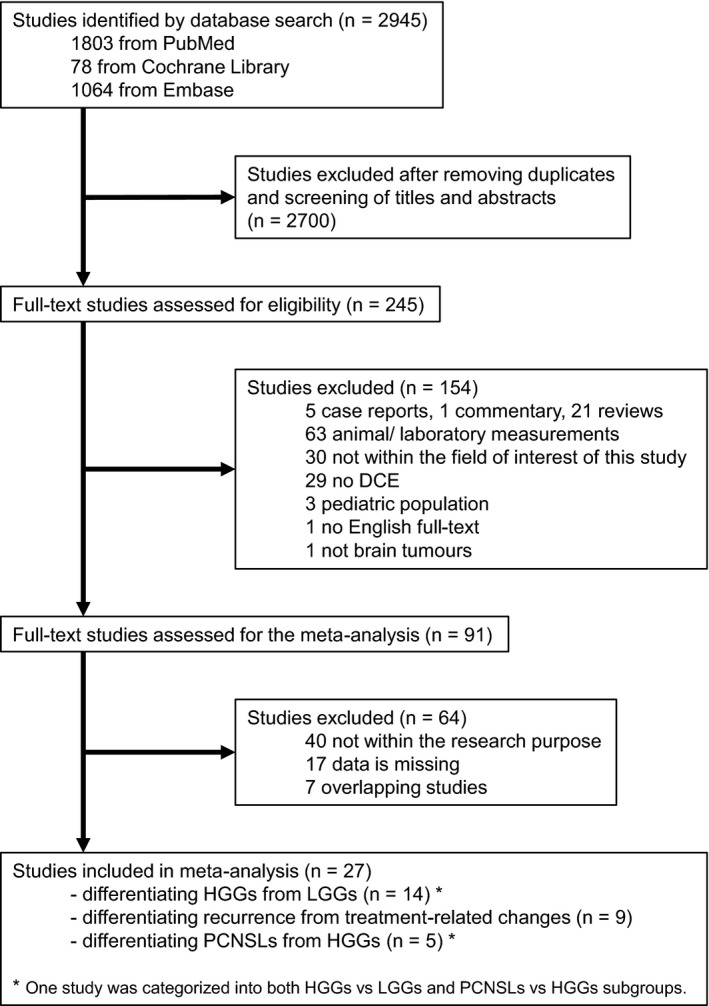
Flowchart describing the study selection process. One study was categorized in two categories (HGGs vs LGGs, PCNSLs vs HGGs). DCE, dynamic contrast‐enhanced; HGG, high‐grade glioma; LGG, low‐grade glioma; PCNSL, primary CNS lymphoma

A total of 2945 articles were confirmed using our electronic database search. After removing duplicate articles and screening the studies titles and abstracts, 245 articles meeting the inclusion criteria underwent full‐text assessment resulting in 27 relevant studies.^12^, ^13^, ^15^, ^18^, ^19^, ^20^, ^23^, ^24^, ^29^, ^30^, ^31^, ^32^, ^33^, ^34^, ^35^, ^36^, ^37^, ^38^, ^39^, ^40^, ^41^, ^42^, ^43^, ^44^, ^45^, ^46^, ^47^ A flowchart of the selection procedure is summarized in Figure 1.

### 3.1 Eligible studies characteristics

We categorized the 27 eligible studies into three groups assessing the role of DCE‐MRI in differentiation: HGGs from LGGs (14 studies^12^, ^18^, ^23^, ^24^, ^29^, ^30^, ^31^, ^32^, ^33^, ^34^, ^35^, ^36^, ^37^, ^38^: 190 LGG and 356 HGG patients), recurrence from treatment‐related changes (9 studies^13^, ^19^, ^39^, ^40^, ^41^, ^42^, ^43^, ^44^, ^45^: 179 patients with relapse and 119 subjects with histologically/clinico‐radiologically verified treatment‐related changes) and PCNSLs from HGGs (5 studies^15^, ^20^, ^34^, ^46^, ^47^: 68 PCNSLs and 156 HGGs patients). One study was categorized into both HGGs vs LGGs and PCNSLs vs HGGs subgroups.^34^ All features of the included studies are demonstrated in Table 1 and Supplementary Material 3. The sensitivity and specificity of each DCE‐derived parameter are listed in Supplementary Material 4.

**Table 1:** The characteristics of the studies included in the meta‐analysis

	High‐grade gliomas vs Low‐grade gliomas	Recurrence vs Treatment‐related changes	Primary central nervous system lymphomas vs High‐grade gliomas
Patients, N	546 (14 studies)	298 (9 studies)	224 (5 studies)
	LGG: 190	Recurrence: 179	PCNSL: 68
	HGG: 356	Treatment‐related change: 119	HGG: 156
DCE model			
Two‐compartment model	437 (12 studies)	183 (6 studies)	182 (4 studies)
Model independent	109 (2 studies)	139 (4 studies)	42 (1 study)
DCE parameters			
*K* ^trans^	352 (8 studies)	183 (6 studies)	125 (2 studies)
v_e_	193 (5 studies)	57 (2 studies)	146 (3 studies)
v_p_	235 (6 studies)	61 (2 studies)	36 (1 study)
Region of interest			
Whole volume	131 (4 studies)	243 (7 studies)	203 (4 studies)
Hot‐spot	415 (10 studies)	55 (2 studies)	21 (1 study)
Country			
China	4 studies		2 studies
USA	3 studies	3 studies	1 study
India	2 studies		
Canada	2 studies		
Korea	1 study	3 studies	1 study
Italy	1 study		
Germany	1 study	2 studies	1 study
Denmark		1 study	

Abbreviations: DCE, dynamic contrast‐enhanced; HGG, high‐grade glioma; LGG, low‐grade glioma; PCNSL, primary CNS lymphoma.

### 3.2 Qualitative assessment

**Figure 2:**
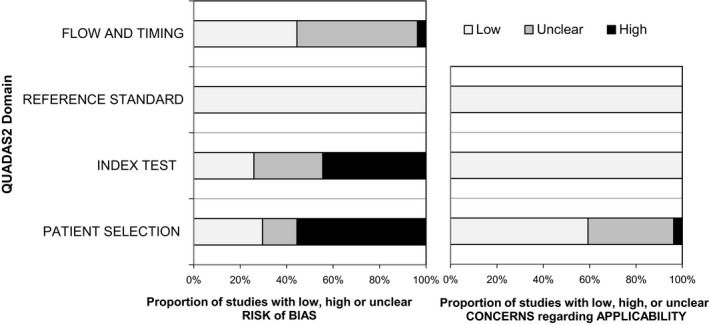
Results of the QUADAS2 quality assessment of the included studies. The risk of bias in four different domains and concerns regarding applicability in three domains are shown

The results of the qualitative assessment are shown in Figure 2. Many studies had high bias in the patient selection and in the conduct or interpretation of the index test because of retrospective study design and a single rater. In more than 10 studies, it was unclear whether radiologists were blinded to histology or whether the interval between MRI and surgery was appropriate.

### 3.3 Diagnostic test accuracy analysis: HGGs vs LGGs

#### 3.3.1 Overall diagnostic accuracy

**Figure 3:**
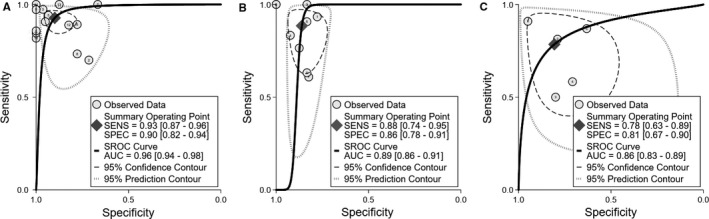
SROC plot of differentiating (A) HGGs from LGGs, (B) recurrence from treatment‐related changes, and (C) PCNSLs from HGGs. AUC, area under the curve; HGG, high‐grade glioma; LGG, low‐grade glioma; PCNSL, primary CNS lymphoma; SENS, sensitivity; SPEC, specificity; SROC, Summary receiver operating characteristic curve

The pooled sensitivity was 0.93 and the pooled specificity was 0.90. Table2 shows PLR, NLR, and DOR. Figure 3A demonstrates the SROC plot with AUC of 0.96, implying high diagnostic accuracy. The sensitivity showed mild heterogeneities (*I* ^2^ = 57.25%), specificity was also heterogeneous (*I* ^2^ = 41.57%). The funnel plot revealed publication bias (*P* = 0.010).

**Table 2:** Results of pooled estimates of studies

	Study number	Sensitivity	Specificity	PLR	NLR	DOR	AUC	*I* ^2^ sensitivity	*I* ^2^ specificity
Diagnostic test accuracy analysis: high‐grade gliomas vs low‐grade gliomas									
All	14	0.93 [0.87‐0.96]	0.90 [0.82‐0.94]	9.2 [5.1‐16.6]	0.08 [0.05‐0.15]	113 [42‐305]	0.96 [0.94‐0.98]	57.25 [31.82‐82.69]	41.57 [4.74‐78.41]
Two‐compartment model	12	0.91 [0.85‐0.95]	0.89 [0.81‐0.94]	8.4 [4.5‐15.6]	0.10 [0.06‐0.17]	82 [31‐216]	0.96 [0.93‐0.97]	47.97 [13.28‐82.67]	40.25 [0.00‐80.85]
model‐independent	2	Study number is too small							
*K^trans^*	8	0.93 [0.84‐0.97]	0.91 [0.82‐0.96]	10.2 [4.9‐21.1]	0.08 [0.04‐0.18]	128 [44‐368]	0.97 [0.95‐0.98]	67.59 [43.47‐91.71]	44.58 [0.00‐89.71]
v_e_	5	0.87 [0.77‐0.93]	0.95 [0.82‐0.99]	18.9 [4.4‐80.1]	0.13 [0.07‐0.25]	141 [22‐883]	0.96 [0.93‐0.97]	35.46 [0.00‐98.77]	43.83 [0.00‐100.00]
v_p_	6	0.83 [0.67‐0.92]	0.91 [0.77‐0.97]	9.0 [3.2‐25.9]	0.19 [0.09‐0.41]	49 [9‐256]	0.94 [0.92‐0.96]	75.46 [55.49‐95.43]	45.74 [0.00‐96.03]
hot‐spot ROI	10	0.95 [0.89‐0.98]	0.90 [0.82‐0.95]	9.3 [5.0‐17.2]	0.05 [0.02‐0.13]	175 [52‐594]	0.96 [0.94‐0.98]	66.15 [43.47‐88.83]	39.94 [0.00‐84.35]
whole volume ROI	4	0.85 [0.73‐0.92]	0.92 [0.68‐0.98]	10.9 [2.2‐53.7]	0.16 [0.08‐0.32]	67 [9‐514]	0.92 [0.90‐0.94]	0.00 [0.00‐100.00]	58.80 [13.48‐100.00]
Diagnostic test accuracy analysis: recurrence vs treatment‐related changes									
All	9	0.88 [0.74‐0.95]	0.86 [0.78‐0.91]	6.4 [3.8‐10.5]	0.13 [0.06‐0.32]	47 [14‐156]	0.89 [0.86‐0.91]	72.77 [54.46‐91.08]	0.00 [0.00‐100.00]
Two‐compartment model	6	0.77 [0.65‐0.86]	0.85 [0.75‐0.92]	5.2 [2.9‐9.3]	0.27 [0.17‐0.44]	19 [8‐47]	0.87 [0.84‐0.90]	45.62 [0.00‐96.02]	0.00 [0.00‐100.00]
model‐independent	4	0.94 [0.86‐0.98]	0.85 [0.74‐0.93]	6.5 [3.4‐12.3]	0.07 [0.03‐0.16]	93 [29‐300]	0.96 [0.94‐0.97]	0.00 [0.00‐100.00]	0.00 [0.00‐100.00]
*K^trans^*	6	0.75 [0.63‐0.84]	0.79 [0.68‐0.87]	3.6 [2.3‐5.8]	0.32 [0.21‐0.49]	11 [5‐25]	0.82 [0.78‐0.85]	40.32 [0.00‐95.54]	0.00 [0.00‐100.00]
v_e_	2	Study number is too small							
v_p_	2	Study number is too small							
hot‐spot ROI	2	Study number is too small							
whole volume ROI	7	0.91 [0.73‐0.97]	0.88 [0.78‐0.93]	7.3 [3.8‐13.8]	0.11 [0.03‐0.34]	68 [14‐328]	0.91 [0.88‐0.93]	76.12 [58.35‐93.89]	0.00 [0.00‐100.00]
Diagnostic test accuracy analysis: primary central nervous system lymphomas vs high‐grade gliomas									
All	5	0.78 [0.63‐0.89]	0.81 [0.67‐0.90]	4.1 [2.1‐7.7]	0.27 [0.14‐0.51]	15 [5‐50]	0.86 [0.83‐0.89]	51.10 [2.09‐100.00]	69.63 [41.11‐98.16]
Two‐compartment model	4	0.75 [0.53‐0.89]	0.83 [0.69‐0.92]	4.5 [2.0‐10.3]	0.30 [0.14‐0.67]	15 [3‐70]	0.86 [0.83‐0.89]	45.87 [0.00‐100.00]	67.52 [32.79‐100.00]
model‐independent	1	Study number is too small							
*K^trans^*	2	Study number is too small							
v_e_	3	Study number is too small							
v_p_	1	Study number is too small							
hot‐spot ROI	1	Study number is too small							
whole tumor ROI	4	0.82 [0.67‐0.91]	0.81 [0.64‐0.91]	4.3 [2.0‐9.2]	0.22 [0.11‐0.47]	19 [5‐77]	0.88 [0.85‐0.90]	50.13 [0.00‐100.00]	77.41 [54.83‐99.99]

The numbers in the parentheses are 95% confidence intervals.

Abbreviations: AUC, area under the curve; DOR, diagnostic odds ratio; NLR, negative likelihood ratio; PLR, positive likelihood ratio; ROI, region of interest.

#### 3.3.2 Subgroup analyses

The results of the subgroup analyses are shown in Table 2. Sensitivity (0.95) was higher for studies with the hot‐spot method of ROI. AUC (0.97) was the highest for the studies that used *K* ^trans^. Heterogeneity was lower for the studies that used v_e_. The model‐independent parameters were not entitled for subgroup meta‐analysis due to the small number of studies.

### 3.4 Diagnostic test accuracy analysis: recurrence vs treatment‐related changes

#### 3.4.1 Overall diagnostic accuracy

The pooled sensitivity was 0.88 and the pooled specificity was 0.86. Table 2 *shows* PLR, NLR, and DOR. Figure 3B exhibits the SROC plot with AUC of 0.89, suggesting moderate diagnostic accuracy. The sensitivity analysis showed substantial heterogeneity (*I* ^2^ = 72.77%) and the specificity analysis presented low heterogeneity (*I* ^2^ = 0.00%).

#### 3.4.2 Subgroup analyses

Table 2 summarizes the results of the subgroup analyses. Sensitivity (0.94) and AUC (0.96) were the highest for studies using model‐independent approaches. The subgroup analysis for the two‐compartment model approach, the model‐independent approach, and *K* ^trans^ estimation had no obvious heterogeneity. Articles with v_e_, v_p_ calculation, and “hot‐spot” ROI placement were not eligible for further subgroup meta‐analysis.

### 3.5 Diagnostic test accuracy analysis: PCNSLs vs HGGs

#### 3.5.1 Overall diagnostic accuracy

The pooled sensitivity and specificity were 0.78 and 0.81, respectively. Table 2 *shows* PLR, NLR, DOR, and AUC. Figure 3C presents the SROC plot with AUC of 0.86, demonstrating moderate diagnostic accuracy. The sensitivity and specificity were characterized by mild heterogeneity (*I* ^2^ = 51.10% and 69.63%, respectively).

#### 3.5.2 Subgroup analyses

The results of the subgroup analyses are detailed in Table 2. We could perform subgroup analysis only for studies with two‐compartment model approaches (N = 4) and whole volume analysis (N = 4).

## 4 DISCUSSION

Our results suggest that DCE‐MRI can stage gliomas into HGGs and LGGs with high diagnostic performance, whereas the accuracy in discriminating between tumor recurrence and unspecific treatment‐induced changes, and between PCNSLs and mimicking HGGs is slightly lower. The overall diagnostic performance results indicate that DCE‐MRI can be successfully utilized in the current neuro‐oncological clinical practice.

Our work adds to the existing literature and a previous systematic review and meta‐analysis, which had compared the diagnostic value of selected advanced MRI techniques, including DCE‐MRI, in brain tumors.^8^, ^48^, ^49^, ^50^ We believe that this is the first meta‐analysis to perform subgroup analyses addressing the type of ROI analysis, the applied pharmacokinetic model, and DCE‐MRI derived parameters. DCE‐MRI as perfusion surrogate measures is relatively understudied because data noise and model fitting instabilities have a remarkable effect on the modeling process.^51^ Parameter values and diagnostic accuracy differ also depending on the methods of ROI selection with the optimal strategy to be still an open debate.

Among the applied ROI methods for stratifying gliomas, “hot‐spot” measurement had higher accuracy than whole volume ROI, in line with the report by Santarosa et al^38^ Although “hot‐spot” is presumed to reflect accurate staging, whole lesion measurement is reproducible, comprehensive but can be time consuming.

To differentiate between recurrence and treatment‐related changes, the model‐independent showed clearly higher sensitivity and AUC than for the 2‐compartment model‐derived perfusion biomarkers, as reported by Hamilton et al^19^ Model‐independent parameters are generally preferred because temporal resolution requirements are relaxed and the potential for fit failure owing to signal noise is irrelevant.^52^

There are some limitations in our study. First, the analysis of studies aiming at grading gliomas revealed publication bias and the composition of the two groups was imbalanced. Most analyses indicated substantial heterogeneity in terms of MR field strength, different types of MR coils, pulse sequence parameters, volume of contrast agent, injection time, which all could affect the outcomes. Some studies performed DCE using only half of contrast agent for DCE‐MRI, followed with DSC‐MRI.^12^, ^38^ ROI methodology, DCE parameters, and DCE models (most studies were on the basis of the two‐compartment Tofts‐Kermode model) also differed substantially prompting us to perform subgroup analyses, which in turn indicated substantial heterogeneity. Model‐independent analysis papers also reported different parameters on each study. The study designs of the included studies revealed only retrospective analyses, lack of consensus and blinding in placing ROIs exposing the studies to substantial bias. Another limitation is the small number studies included in subgroup analyses, and we acknowledge that further studies are needed for adding credibility. Last but not least, in the era of integrated histomolecular glioma classification, there was insufficient number of studies which evaluated the diagnostic accuracy of molecular subtype using DCE‐MRI.^53^

In conclusion, our results suggest that DCE‐MRI is a promising noninvasive imaging method that has good accuracy in diagnosing different types of brain tumors. Specifically, DCE‐MRI has high diagnostic performance in stratifying gliomas in high‐ or low‐grade, and moderate diagnostic accuracy in differentiating recurrence from treatment‐related changes, and PCNSLs from HGGs. Significant efforts for the standardization of the acquisition parameters and the postprocessing should be, however, intensely made.

## CONFLICT OF INTEREST

None declared.

## AUTHOR CONTRIBUTIONS

SO, AU, JU, ST, and SB were involved in conception and design. SO, AU, IL, JU, ML, SMH, ES, ST, and SB were involved in acquisition of data (screening and data extraction). SO, ARG, JPG, XG, and SB were involved in statistical analysis and interpretation of data. All authors were involved in drafting, editing and critical revision for important intellectual content. All authors gave final approval of the version to be published.
