# Supplementary material for: Diagnostic accuracy of dynamic contrast‐enhanced perfusion MRI in stratifying gliomas: A systematic review and meta‐analysis
Source: Cancer Med. 2019 Aug 7;8(12):5564–73. doi: 10.1002/cam4.2369 (PMC6745862; doi:10.1002/cam4.2369)
Supplement: Supplementary file 1 [file CAM4-8-5564-s001.docx]

Supplementary material 1

Search strategy in PubMed:

((((((((((((((brain tumour[tw]) OR brain tumor[tw]) OR glioma[tw]) OR brain metastasis[tw]) OR brain neoplasm[tw]) OR brain cancer[tw]) OR glioblastoma[tw]) OR brain neoplasm[MeSH Terms]) OR glioma[MeSH Terms]) AND (((((((((((((((((magnetic resonance imaging[MeSH Terms] AND perfusion[tw]) OR DCE[tw]) OR DCE-MRI[tw]) OR dynamic contrast-enhanced[tw]) OR dynamic contrast enhanced[tw]) OR dynamic MRI[tw]) OR T1 perfusion[tw]) OR volume transfer[tw]) OR extravascular extracellular volume) OR fractional plasma volume) OR permeability[tw]) OR interstitial volume[tw]) OR Ktrans) OR pharmacokinetic MRI) OR pharmacokinetic modeling[tw]) OR pharmacokinetic modelling[tw]) AND ("1990/01/01"[PDat] : "2017/06/08"[PDat]) AND Humans[Mesh]))))))

Search strategy in the Cochrane Library:

#1 "brain tumour" or "brain tumor" or glioma or (brain next metastasis) or (brain next neoplasm) or "brain cancer"

#2 MeSH descriptor: [Brain Neoplasms] explode all trees

#3 MeSH descriptor: [Glioma] explode all trees

#4 MeSH descriptor: [Oligodendroglioma] explode all trees

#5 MeSH descriptor: [Astrocytoma] explode all trees

#6 MeSH descriptor: [Neoplasm Metastasis] explode all trees

#7 #1 or #2 or #3 or #4 or #5 or #6

#8 DCE MRI or DCE or "dynamic contrast-enhanced" or dynamic MRI or T1 perfusion or DCE MRI or "volume transfer" or "extravascular extracellular volume" or "fractional plasma volume" or permeability or "interstitial volume" or Ktrans or (pharmacokinetic next MRI) or "pharmacokinetic modeling" or "pharmacokinetic modelling"

#9 MeSH descriptor: [Magnetic Resonance Imaging] explode all trees

#10 perfusion

#11 #8 or (#9 and #10)

#12 #7 and #11

Publication Year from 1990 to 2017

Search strategy in Embase:

1. exp glioma/

2. exp astrocytoma/

3. exp oligodendroglioma/

4. exp metastasis/

5. exp brain tumor/

6. "brain tumour".ti,ab,kw.

7. "brain tumor".ti,ab,kw.

8. glioma.ti,ab,kw.

9. "cerebr*".ti,ab,kw.

10. ("cerebr*" adj5 metastas#s).ti,ab,kw.

11. (brain adj5 metastas#s).ti,ab,kw.

12. (brain adj5 cancer).ti,ab,kw.

13. (brain adj5 neoplasm).ti,ab,kw.

14. glioblastoma.ti,ab,kw.

15. brain.ti,ab,kw.

16. 9 or 15

17. 4 and 16

18. 1 or 2 or 3 or 5 or 6 or 7 or 8 or 10 or 11 or 12 or 13 or 14 or 17

19. DCE.ti,ab,kw.

20. DCE-MRI.ti,ab,kw.

21. "dynamic contrast enhanced".ti,ab,kw.

22. dynamic MRI.ti,ab,kw.

23. T1 perfusion.ti,ab,kw.

24. volume transfer.ti,ab,kw.

25. extravascular extracellular volume.ti,ab,kw.

26. fractional plasma volume.ti,ab,kw.

27. permeability.ti,ab,kw.

28. interstitial volume.ti,ab,kw.

29. ktrans.ti,ab,kw.

30. pharmacokinetic MRI.ti,ab,kw.

31. pharmacokinetic model?ing.ti,ab,kw.

32. 19 or 20 or 21 or 22 or 23 or 24 or 25 or 26 or 27 or 28 or 29 or 30 or 31

33. exp nuclear magnetic resonance imaging/

34. perfusion.ti,ab,kw.

35. 33 and 34

36. 32 or 35

37. 18 and 36

38. limit 37 to (human and yr="1990 -Current" and (adult <18 to 64 years> or aged <65+ years>))
