# Supplementary material for: Diagnostic accuracy of dynamic contrast‐enhanced perfusion MRI in stratifying gliomas: A systematic review and meta‐analysis
Source: Cancer Med. 2019 Aug 7;8(12):5564–73. doi: 10.1002/cam4.2369 (PMC6745862; doi:10.1002/cam4.2369)
Supplement: Supplementary file 2 [file CAM4-8-5564-s002.docx]

Supplementary material 2. Risk of bias and applicability judgements (QUADAS-2).

| **Domain** | **Signaling questions** | **Risk of bias (low, high, unclear)** | **Concerns regarding applicability (low, high, unclear)** |
| --- | --- | --- | --- |
| **1. Patient selection** | Were adult (> 18 years old) patients more than 90% of patients? | Could the selection of patients have introduced bias? | Is there concern that the included patients do not match the review question? |
|  | Did the study avoid inappropriate exclusions? |  |  |
|  | Prospective study or retrospective study? |  |  |
| **2. Index test** | Was DCE model 2-compartment model? | Could the conduct or interpretation of the index test have introduced bias? | Is there concern that the index test, its conduct, or interpretation differ from the review question? |
|  | Were the index test results interpreted without knowledge of the results of the reference standard? |  |  |
|  | Was ROI selected by two evaluators? |  |  |
| **3. Reference standard** | Is the reference standard likely to correctly classify the target condition? | Could the reference standard, its conduct, or its interpretation have introduced bias? | Is there concern that the target condition as defined by the reference standard does not match the review question? |
|  | Were the reference standard results interpreted without knowledge of the results of the index test? |  |  |
| **4. Flow and timing** | Was there an appropriate interval between index test(s) and reference standard? | Could the patient flow have introduced bias? |  |
|  | Did all patients receive a reference standard? |  |  |
|  | Were all patients included in the analysis? |  |  |

Abbreviations: DCE = dynamic contrast-enhanced, ROI = region of interest.
