# Supplementary material for: Diagnostic accuracy of dynamic contrast‐enhanced perfusion MRI in stratifying gliomas: A systematic review and meta‐analysis
Source: Cancer Med. 2019 Aug 7;8(12):5564–73. doi: 10.1002/cam4.2369 (PMC6745862; doi:10.1002/cam4.2369)
Supplement: Supplementary file 3 [file CAM4-8-5564-s003.docx]

Supplementary material 3

The characteristics of the included studies for differentiating between high grade gliomas (HGGs) and low grade gliomas (LGGs)

| **Study (first author, year of publication)** | **Age mean±SD  (range)** | **Study design** | **Country** | **Model** | **Deconvolution with AIF** | **Was DCE followed with DSC ?** | **ROI** | **Field strength** | **No. of total patients** | **Histology** | **MRI parameters** | **Sensitivity (%)** | **Specificity (%)** |
| --- | --- | --- | --- | --- | --- | --- | --- | --- | --- | --- | --- | --- | --- |
| Arevalo 2015 ^18^ | 54 | Retrospective | USA | The 2-compartment pharmacokinetic model by Tofts | AIF (MCA) | No | whole tumor | 1.5T | 63 | WHO II 20 WHO III 10 WHO IV 33 | mean _r_v_p_ | 90.7 | 95.0 |
|  |  |  |  |  |  |  |  |  |  |  | mean _r_*K^trans^* | 79.1 | 95.0 |
|  |  |  |  |  |  |  |  |  |  |  | max _r_v_p_ | 83.7 | 95.0 |
|  |  |  |  |  |  |  |  |  |  |  | max _r_*K^trans^* | 86.0 | 85.0 |
| Arevalo 2016 ^29^ | 45.2 (39-82) | Retrospective | USA | The 2-compartment pharmacokinetic model by Tofts | AIF (MCA) | No | whole tumor | 1.5/3T | 24 | WHO II 14 WHO III 10 | mean v_p_ | 70.0 | 70.0 |
| Jia 2013 ^30^ | 47±12 (13–74) | Unclear | China | The Tofts and Kermode pharmacokinetic model | AIF (ICA) | No | hot spot | 3T | 65 | WHO II 27 WHO III 38 | mean *K^trans^* | 97.4 | 96.3 |
|  |  |  |  |  |  |  |  |  |  |  | mean v_e_ | 94.7 | 100.0 |
| Jung 2014 ^31^ | 49.75  (25–72) | Retrospective | Korea | The 2-compartment pharmacokinetic model by Tofts and Kermode | AIF (intracranial tumor supplying artery near the tumor) | No | whole tumor | 3T | 28 | WHO II 7 WHO III 8 WHO IV 13 | 98%_tile_ *K^trans^* | 76.2 | 100.0 |
|  |  |  |  |  |  |  |  |  |  |  | 90%_tile_ v_e_ | 85.7 | 100.0 |
|  |  |  |  |  |  |  |  |  |  |  | 84%_tile_ v_p_ | 61.9 | 100.0 |
| Li 2015 ^32^ | 42.6 ± 14.3  (12-69) | Retrospective | China | The 2-compartment pharmacokinetic model by Tofts and Kermode | Unclear | No | hot spot | 3T | 32 | WHO II 15 WHO III 8  WHO IV 9 | mean *K^trans^* | 94.1 | 93.3 |
|  |  |  |  |  |  |  |  |  |  |  | mean v_e_ | 92.9 | 91.7 |
| Nguyen 2012 ^33^ | Unclear | Prospective | Canada | The 2-compartment extended Tofts model | VIF (SSS) | No | hot spot | 1.5/3T | 46 | WHO II 9 WHO III 9 WHO IV 28 | median *K^trans^* | 89.0 | 75.0 |
|  |  |  |  |  |  |  |  |  |  |  | median v_p_ | 68.0 | 89.0 |
| Nguyen 2015 ^12^ | 57  (95％CI, 53-62) | Prospective | Canada | The 2-compartment extended Tofts model using phase-derived VIF with bookend T1 measurements and using magnitude-derived VIF without T1 correction | VIF (SSS) | Yes | hot spot | 3T | 48 | WHO II 9 WHO III 11 WHO IV 28 | median *K^trans^* (phase-derived) | 100.0 | 67.0 |
|  |  |  |  |  |  |  |  |  |  |  | median *K^trans^* (magnitude-derived) | 79.0 | 78.0 |
|  |  |  |  |  |  |  |  |  |  |  | median v_p_ (phase-derived) | 90.0 | 89.0 |
|  |  |  |  |  |  |  |  |  |  |  | median v_p_ (magnitude-derived) | 79.0 | 89.0 |
| Zhao 2015 ^34^ | 46 | Prospective | China | The 2-compartment model | AIF | No | 6-10 ROIs within tumor parenchyma | 3T | 24 | WHO I+II 9 WHO III+IV 15 | mean v_e_ | 76.0 | 79.0 |
| Jain 2015 ^35^ | Unclear | Prospective | India | Quantitative analysis of the concentrationetime curve | Not required | No | hot spot | 3T | 53 | WHO I 2  WHO II 15 WHO III 9 WHO IV 27 | mean rCBV | 97.2 | 100.0 |
| Ludemann 2000 ^23^ | (12-78) | Unclear | Germany | The modified pharmacokinetic model | Unclear | No | whole tumor | 1.5T | 16 | WHO II 5 WHO III 5 WHO IV 6 | Arith.mean (V_b_/V_t_) | 81.8 | 100.0 |
|  |  |  |  |  |  |  |  |  |  |  | Quadr. Mean (V_b_/V_t_) | 72.7 | 100.0 |
| Roberts 2000 ^24^ | (14-79) | Unclear | USA | The 2-compartment model | VIF (SSS) | No | hot spot | 1.5T | 21 | WHO II 8 WHO III 7 WHO IV 6 | fBV | 61.5 | 87.5 |
|  |  |  |  |  |  |  |  |  |  |  | k | 84.6 | 100.0 |
|  |  |  |  |  |  |  |  |  |  |  | k^ps^ | 84.6 | 87.5 |
| Jia 2012 ^36^ | 45±14 (13-74) | Unclear | China | Tofts and Kermode pharmacokinetic model | Unclear | No | hot spot | 3T | 44 | WHO II 17 WHO III 2 WHO IV 25 | mean *K^trans^* | 88.9 | 82.4 |
|  |  |  |  |  |  |  |  |  |  |  | mean v_e_ | 81.5 | 94.1 |
| Roy 2013 ^37^ | (14-69) | Unclear | India | Quantitative analysis of the concentration-time curve | Not required | No | hot spot | 3T | 56 | WHO I+II 24 WHO III+IV 32 | mean rCBV | 100.0 | 88.0 |
| Santarosa2016 ^38^ | 55.4 (22-79) | Prospective | Italy | The extended 2-compartment pharmacokinetic Tofts and Kermode’s model | VIF (SSS) | Yes | hot spot | 3T | 26 | WHO II 9 WHO III 4 WHO IV 13 | v_p_ max | 100.0 | 100.0 |
|  |  |  |  |  |  |  |  |  |  |  | *K^trans^* max | 100.0 | 100.0 |

The characteristics of the included studies for differentiating between recurrence and treatment-related changes

| **Study (first author, year of publication)** | **Age mean±SD  (range)** | **Study design** | **Country** | **Model** | **Deconvolution with AIF** | **Was double perfusion technique used or not?** | **ROI** | **Field strength** | **No. of total patients** | **No. of recurrence** | **No. of treatment-related changes** | **Histology** | **MRI parameters** | **Sensitivity (%)** | **Specificity (%)** |
| --- | --- | --- | --- | --- | --- | --- | --- | --- | --- | --- | --- | --- | --- | --- | --- |
| Hamilton 2015 ^19^ | 51±9 | Retrospective | USA | The 2-compartment generalized kinetic model | VIF (MCA or SSS) | Yes | hot spot | 3T | 24 | 15 | 9 | WHO II 1 WHO III 8 WHO IV 15 | mean *K^trans^* | 80 | 78 |
|  |  |  |  |  |  |  |  |  |  |  |  |  | max *K^trans^* | 80 | 78 |
|  |  |  |  |  |  |  |  |  |  |  |  |  | mean Kep | 47 | 78 |
|  |  |  |  |  |  |  |  |  |  |  |  |  | mean v_p_ | 71 | 89 |
|  |  |  |  |  |  |  |  |  |  |  |  |  | max v_p_ | 73 | 67 |
|  |  |  |  |  |  |  |  |  |  |  |  |  | mean v_e_ | 80 | 78 |
|  |  |  |  | The model-independent analysis |  |  |  |  |  |  |  |  | short AUC | 93 | 67 |
|  |  |  |  |  |  |  |  |  |  |  |  |  | intermediate AUC | 93 | 67 |
|  |  |  |  |  |  |  |  |  |  |  |  |  | delayed short AUC | 93 | 78 |
| Yun 2015 ^39^ | 54.6  (28-82) | Prospective | Korea | The 2-compartment pharmacokinetic model by Tofts and Kermode | AIF (intracranial tumor supplying artery near the tumor) | No | whole tumor | 3T | 33 | 17 | 16 | WHO IV 33 | mean *K^trans^* | 59 | 94 |
|  |  |  |  |  |  |  |  |  |  |  |  |  | 10%_tile_ *K^trans^* | 82 | 75 |
|  |  |  |  |  |  |  |  |  |  |  |  |  | mean v_e_ | 88 | 56 |
|  |  |  |  |  |  |  |  |  |  |  |  |  | 5%_tile_ v_e_ | 76 | 88 |
| Bisdas 2011 ^40^ | unclear | Prospective | Germany | The 2-compartment model | AIF | No | whole tumor | 3T | 18 | 12 | 6 | WHO III+IV 18 | median *K^trans^* | 100 | 83 |
|  |  |  |  |  |  |  |  |  |  |  |  |  | median iAUC | 71 | 71 |
| Thomas 2015 ^41^ | 63  (37-87) | Retrospective | USA | The 2-compartment model proposed by Tofts | AIF (a suitable artery to typify the input function curve and the concentration-time curve) | No | whole tumor | 1.5/3T | 37 | 24 | 13 | WHO IV 37 | mean _r_*K^trans^* | 69 | 79 |
|  |  |  |  |  |  |  |  |  |  |  |  |  | mean v_p_ | 85 | 79 |
|  |  |  |  |  |  |  |  |  |  |  |  |  | 90%_tile_ v_p_ | 85 | 92 |
| Shin 2014 ^13^ | 54.5 ±11.9  (27-72) | Retrospective | Korea | The 2-compartment pharmacokinetic model by Tofts | AIF | Yes | hot spot | 3T | 31 | 19 | 12 | WHO II 4 WHO III 7 WHO IV 20 | mean _r_*K^trans^* | 61.11 | 80 |
|  |  |  |  |  |  |  |  |  |  |  |  |  | mean _r_ iAUC | 66.67 | 70 |
| Suh 2013 ^42^ | 51.2  (25-69) | Retrospective | Korea | Time–signal intensity curve | Not required | No | whole tumor | 3T | 79 | 43 | 36 | WHO IV 79 | AUCR50 | 87.2 | 83.1 |
|  |  |  |  |  |  |  |  |  |  |  |  |  | AUCR75 | 82.6 | 81.1 |
|  |  |  |  |  |  |  |  |  |  |  |  |  | AUCR90 | 89.6 | 81.7 |
|  |  |  |  |  |  |  |  |  |  |  |  |  | AUCRmode | 73.1 | 79.7 |
|  |  |  |  |  |  |  |  |  |  |  |  |  | mAUCRH | 90.1 | 82.9 |
| Narang 2011 ^43^ | treatment related changes51.4 (18-70) recurrence 52.3 (28-66) | Retrospective | USA | Time–signal intensity curve | Not required | No | whole tumor | 3T | 22 | 15 | 7 | WHO III 5 WHO IV 17 | MSIVP | 100 | 100 |
| Seeger 2013 ^44^ | 53.6 ± 13.6 | Retrospective | Germany | The modified Tofts-Kermode model | Unclear | Yes | whole tumor | 1.5T | 40 | 23 | 17 | WHO III+IV 40 | max *K^trans^* | 61.9 | 80 |
| Larsen 2013 ^45^ | 28-76 | Prospective | Denmark | Patlak plot model-free deconvolution with Tikhonov regularization | AIF (ICA) | No | whole tumor | 3T | 14 | 11 | 3 | WHO II 1 WHO III+IV 13 | CBV | 100 | 100 |

The characteristics of the included studies for differentiating between primary central nervous system lymphomas (PCNSLs) and HGGs

| **Study (first author, year of publication)** | **Age mean±SD  (range)** | **Study design** | **Country** | **Model** | **Deconvolution with AIF** | **Was double perfusion technique used or not?** | **ROI** | **Field strength** | **No. of total patients** | **No. of PCNSL** | **No. of HGG** | **MRI parameters** | **Sensitivity (%)** | **Specificity (%)** |
| --- | --- | --- | --- | --- | --- | --- | --- | --- | --- | --- | --- | --- | --- | --- |
| Kickingereder 2014 ^15^ | Unclear | Retrospective | Germany | The Tofts-Kermode model | AIF | No | whole tumor | 3T | 71 | 11 | 60  (WHO IV) | median *K^trans^* | 90.9 | 95 |
|  |  |  |  |  |  |  |  |  |  |  |  | median Kep | 90.9 | 78.3 |
|  |  |  |  |  |  |  |  |  |  |  |  | median v_e_ | 63.6 | 76.7 |
| Lu 2016 ^46^ | GBM 52 (29-79) PCNSL 61 (45-75) | Retrospective | China | The modified Tofts model | VIF (SSS) | No | whole tumor | 3T | 54 | 16 | 38  (WHO IV) | mean *K^trans^* | 81.2 | 79 |
|  |  |  |  |  |  |  |  |  |  |  |  | mean v_e_ | 81.2 | 65.8 |
| Zhao 2015 ^34^ | 46 | Prospective | China | The 2-compartment model | AIF | No | 6-10 ROIs within tumor parenchyma | 3T | 21 | 6 | 15  (WHO III+IV) | mean v_e_ | 53 | 79 |
| Lin 2017 ^20^ | GBM 68.6 (46-88)  PCNSL 68.7 (47-84) | Retrospective | USA | The 2-compartment pharmacokinetic model by Tofts | AIF | No | whole tumor | 1.5/3T | 36 | 12 | 24  (WHO IV) | 90% _tile_ _r_v_p_ | 58 | 72 |
| Choi 2017 ^47^ | 59.7±2.1 | Retrospective | Korea | Time signal intensity curve | Not required | No | whole tumor | 3T | 42 | 23 | 19  (WHO IV) | mean IAUC30 | 65.2 | 78.9 |
|  |  |  |  |  |  |  |  |  |  |  |  | 90%_tile_ IAUC30 | 87 | 63.2 |
|  |  |  |  |  |  |  |  |  |  |  |  | 90%_tile_ IAUC60 | 69.6 | 68.4 |
|  |  |  |  |  |  |  |  |  |  |  |  | 90%_tile_ IAUC90 | 69.6 | 68.4 |

Abbreviations: AIF = arterial input function, VIF = venous input function, DCE = dynamic contrast-enhanced, DSC = dynamic susceptibility contrast-enhanced, ROI = region of interest, MCA = middle cerebral artery, ICA = internal carotid artery, SSS = superior sagittal sinus, AUC = area under the curve
