# Supplementary material for: Diagnostic accuracy of dynamic contrast‐enhanced perfusion MRI in stratifying gliomas: A systematic review and meta‐analysis
Source: Cancer Med. 2019 Aug 7;8(12):5564–73. doi: 10.1002/cam4.2369 (PMC6745862; doi:10.1002/cam4.2369)
Supplement: Supplementary file 4 [file CAM4-8-5564-s004.docx]

Supplementary material 4

The sensitivity and specificity of each DCE (dynamic contrast-enhanced)-MRI derived parameter for differentiating between high grade gliomas (HGGs) and low grade gliomas (LGGs)

| **MRI parameters** | **Study (first author, year of publication)** | **Sensitivity (%)** | **Specificity (%)** |
| --- | --- | --- | --- |
| mean Ktrans | Jia 2013 | 97.4 | 96.3 |
|  | Li2015 | 94.1 | 93.3 |
|  | Jia 2012 | 88.9 | 82.4 |
| median Ktrans | Nguyen 2012 | 89.0 | 75.0 |
|  | Nguyen 2015 (magnitude-derived VIF) | 79.0 | 78.0 |
|  | Nguyen 2015 (phase-derived VIF) | 100.0 | 67.0 |
| mean rKtrans | Arevalo 2015 | 79.1 | 95.0 |
| max Ktrans | Santarosa2016 | 100.0 | 100.0 |
| max rKtrans | Arevalo 2015 | 86.0 | 85.0 |
| 98%_tile_ Ktrans | Jung 2014 | 76.2 | 100.0 |
| mean Ve | Jia 2013 | 94.7 | 100.0 |
|  | Li2015 | 92.9 | 91.7 |
|  | Jia 2012 | 81.5 | 94.1 |
|  | Zhao 2015 | 76.0 | 79.0 |
| 90%_tile_ Ve | Jung 2014 | 85.7 | 100.0 |
| mean Vp | Arevalo 2016 | 70.0 | 70.0 |
| median Vp | Nguyen 2012 | 68.0 | 89.0 |
|  | Nguyen 2015 (phase-derived VIF) | 90.0 | 89.0 |
|  | Nguyen 2015 (magnitude-derived VIF) | 79.0 | 89.0 |
| 84%_tile_ Vp | Jung 2014 | 61.9 | 100.0 |
| max Vp | Santarosa2016 | 100.0 | 100.0 |
| mean rVp | Arevalo 2015 | 90.7 | 95.0 |
| max rVp | Arevalo 2015 | 83.7 | 95.0 |
| mean rCBV | Jain 2015 | 97.2 | 100.0 |
|  | Roy 2013 | 100.0 | 88.0 |
| Arith.mean (Vb/Vt) | Ludemann 2000 | 81.8 | 100.0 |
| Quadr. mean (Vb/Vt) | Ludemann 2000 | 72.7 | 100.0 |
| fBV | Roberts 2000 | 61.5 | 87.5 |
| k | Roberts 2000 | 84.6 | 100.0 |
| kps | Roberts 2000 | 84.6 | 87.5 |

The sensitivity and specificity of each DCE (dynamic contrast-enhanced)-MRI derived parameter for differentiating between recurrence and treatment-related changes

| **MRI parameters** | **Study (first author, year of publication)** | **Sensitivity (%)** | **Specificity (%)** |
| --- | --- | --- | --- |
| mean Ktrans | Hamilton 2015 | 80 | 78 |
|  | Yun 2015 | 59 | 94 |
| median Ktrans | Bisdas 2011 | 100 | 83 |
| mean rKtrans | Thomas 2015 | 69 | 79 |
|  | Shin 2014 | 61.11 | 80 |
| 10%_tile_ Ktrans | Yun 2015 | 82 | 75 |
| max Ktrans | Hamilton 2015 | 80 | 78 |
|  | Seeger 2013 | 61.9 | 80 |
| mean Ve | Hamilton 2015 | 80 | 78 |
|  | Yun 2015 | 88 | 56 |
| 5%_tile_ Ve | Yun 2015 | 76 | 88 |
| mean Vp | Hamilton 2015 | 71 | 89 |
|  | Thomas 2015 | 85 | 79 |
| max Vp | Hamilton 2015 | 73 | 67 |
| 90%_tile_ Vp | Thomas 2015 | 85 | 92 |
| mean Kep | Hamilton 2015 | 47 | 78 |
| AUCR50 | Suh 2013 | 87.2 | 83.1 |
| AUCR75 | Suh 2013 | 82.6 | 81.1 |
| AUCR90 | Suh 2013 | 89.6 | 81.7 |
| AUCRmode | Suh 2013 | 73.1 | 79.7 |
| mAUCRH | Suh 2013 | 90.1 | 82.9 |
| delayed short AUC | Hamilton 2015 | 93 | 78 |
| intermediate AUC | Hamilton 2015 | 93 | 67 |
| short AUC | Hamilton 2015 | 93 | 67 |
| mean r iAUC | Shin 2014 | 66.67 | 70 |
| median iAUC | Bisdas 2011 | 71 | 71 |
| CBV | Larsen 2013 | 100 | 100 |
| MSIVP | Narang 2011 | 100 | 100 |

The sensitivity and specificity of each DCE (dynamic contrast-enhanced)-MRI derived parameter for differentiating between primary central nervous system lymphomas (PCNSLs) and HGGs

| **MRI parameters** | **Study (first author, year of publication)** | **Sensitivity (%)** | **Specificity (%)** |
| --- | --- | --- | --- |
| mean Ktrans | Lu 2016 | 81.2 | 79 |
| median Ktrans | Kickingereder 2014 | 90.9 | 95 |
| mean Ve | Lu 2016 | 81.2 | 65.8 |
|  | Zhao 2015 | 53 | 79 |
| median Ve | Kickingereder 2014 | 63.6 | 76.7 |
| 90%_tile_ rVp | Lin 2017 | 58 | 72 |
| median Kep | Kickingereder 2014 | 90.9 | 78.3 |
| 90%_tile_ IAUC30 | Choi 2017 | 87 | 63.2 |
| 90%_tile_ IAUC60 | Choi 2017 | 69.6 | 68.4 |
| 90%_tile_ IAUC90 | Choi 2017 | 69.6 | 68.4 |
| mean IAUC30 | Choi 2017 | 65.2 | 78.9 |
